# Teprotumumab in the management of thyroid eye disease mechanistic insights and adverse reactions: a comprehensive review

**DOI:** 10.3389/fendo.2025.1480195

**Published:** 2025-05-27

**Authors:** Zhibin Xu, Nan Hu, Qitao Chen, Yue Zhou, Yunye Huang, Songhao Hu, Haoru Jin, Yubo Tang

**Affiliations:** ^1^ Department of Clinical Research, First Affiliated Hospital of Jinan University, Guangzhou, China; ^2^ Clinical Research Center (CRC), The First Affiliated Hospital of Jinan University, Guangzhou, China; ^3^ Department of Pharmacy, Sanming Integrative Medicine Hospital, Sanming, Fujian, China; ^4^ Department of Otolaryngology, The First Affiliated Hospital of Jinan University, Guangzhou, China; ^5^ Department of Gastrointestinal Surgery, The First Affiliated Hospital of Jinan University, Guangzhou, China; ^6^ Department of Thoracic Surgery, State Key Laboratory of Respiratory Disease, National Clinical Research Center for Respiratory Disease, Guangzhou Institute of Respiratory Health, First Affiliated Hospital of Guangzhou Medical University, Guangzhou, China; ^7^ Department of Pharmacy, The First Affiliated Hospital, Sun Yat-sen University, Guangzhou, China

**Keywords:** teprotumumab, thyroid eye disease (TED), adverse reactions, hearing impairment, hyperglycemia, inflammatory bowel disease (IBD)

## Abstract

Teprotumumab has shown significant efficacy in treating Thyroid Eye Disease (TED), but its adverse effects require careful management. Key reactions include hearing impairment, hyperglycemia, and potential exacerbation of pre-existing inflammatory bowel disease (IBD). Hearing impairment, likely due to inhibition of the insulin-like growth factor 1 receptor (IGF-1R), manifests as more severe sensorineural changes. Hyperglycemia results from disrupted growth hormone feedback and may be worsened by prior glucocorticoid use. Although teprotumumab does not appear to induce new diabetes cases, it can exacerbate existing hyperglycemia. Cognitive issues, infusion reactions, and other adverse effects, such as muscle cramps and weight loss, have also been observed. Management requires careful patient screening, particularly for those with histories of hearing loss, diabetes, or IBD. Further research is essential to elucidate the underlying mechanisms of these adverse effects and develop targeted preventive strategies to improve the safety and efficacy of teprotumumab in clinical practice.

## Highlights

- Teprotumumab is an effective treatment for TED, but it’s associated with notable adverse effects that require careful management.
- Hearing impairment, a significant adverse effect, is likely linked to IGF-1R inhibition, which disrupts crucial cochlear cell survival pathways. The high prevalence of pre-existing hearing deficits in TED patients also complicates interpretation of this risk.
- Hyperglycemia may occur, likely due to disrupted feedback inhibition on growth hormone, leading to increased glucose production and insulin resistance, particularly in patients with pre-existing insulin resistance or diabetes. Prior use of glucocorticoids in TED patients may further contribute to glucose dysregulation.
- Speculative evidence suggests that teprotumumab may exacerbate pre-existing inflammatory bowel disease (IBD), though this association relies on anecdotal reports rather than controlled studies.
- Additional adverse effects include muscle cramps, infusion reactions, and cognitive issues, although these are less clearly linked to teprotumumab and require further investigation.
- Multidisciplinary collaboration and thorough patient screening are essential.
- Close monitoring and preemptive measures are crucial for safe treatment.

## 1 Introduction

Thyroid Eye Disease (TED) is an autoimmune condition and one of adults’ most prevalent orbital diseases. It is frequently associated with Graves’ disease (GD), accounting for approximately 80% of cases, but can also occur in euthyroid individuals (10%) and patients with Hashimoto’s thyroiditis or thyroid cancer (10%) (1). The primary clinical features of TED include exophthalmos, conjunctival edema, and diplopia. Severe cases may involve optic neuropathy or corneal ulcers, potentially leading to vision impairment or blindness. Current treatment strategies lack specificity and primarily focus on symptomatic relief, rendering TED a particularly challenging condition to manage in ophthalmology. On January 21, 2020, teprotumumab received approval from the U.S. Food and Drug Administration (FDA) to treat TED (2) and was granted orphan drug status. This approval was based on significant efficacy in phase II and III randomized clinical trials (RCTs) involving patients with moderate-to-severe active TED (3, 4).

Teprotumumab is a human monoclonal antibody that binds with high affinity and specificity to the extracellular domain of the IGF-1R (5, 6). Upon binding to IGF-1R, teprotumumab induces internalization and degradation of the antibody-receptor complex. Due to the structural and functional relationship between IGF-1R and the thyroid-stimulating hormone receptor (TSHR), teprotumumab also inhibits the TSHR signaling pathway, possibly contributing to its therapeutic effects in TED. Notably, Graves’ disease serves as a background autoimmune condition in over 90% of TED patients, and studies have observed that uncontrolled Graves’ disease is associated with higher rates and greater severity of TED. Furthermore, the potential cross-linking between IGF-1R, TSHR, and insulin receptors suggests that the mechanisms of action may extend beyond simple IGF-1R antagonism, incorporating a range of downstream effects and alternate pathways under investigation. This blocks harmful immune proteins (autoantibodies) from attacking orbital fibroblasts, which are key drivers of TED symptoms. Furthermore, it reduces the production of hyaluronic acid, a gel-like molecule that contributes to swelling, and inflammatory signaling molecules (cytokines), thereby controlling the pathological immune response in TED (7, 8). Recent studies have indicated that teprotumumab may also have a blocking effect on the IGF-2 receptor (IGF-2R), although current research on this is limited (7). Most of the studies have focused on the drug’s effect on IGF-1R, and its effects on IGF-2R are still under investigation. This mechanism reduces hyaluronic acid production and cytokine stimulation, effectively halting the pathological immune response in active TED (9). However, despite its promising therapeutic effects, the safety profile of teprotumumab must be carefully considered. While most adverse reactions are mild and manageable, some potential side effects, such as muscle cramps, infusion reactions, and thyroid dysfunction, raise concerns that need to be addressed.

While teprotumumab’s adverse reactions are generally mild and manageable, a comprehensive understanding of these side effects, their underlying mechanisms, and preventive strategies is crucial for optimizing patient outcomes. Most current studies have focused on the therapeutic efficacy of teprotumumab, with relatively few investigations addressing the adverse reactions and their underlying mechanisms. This review aims to bridge this gap by providing a comprehensive analysis of both the mechanistic pathways and clinical safety concerns of teprotumumab in TED treatment. Specifically, we explore how IGF-1R inhibition affects TED pathophysiology and discuss the associated adverse effects, including their mechanisms, incidence, and management strategies.

To achieve this, we conducted a literature search using PubMed, Web of Science, and Scopus databases up to August 2024. The search terms included “Teprotumumab,” “Thyroid Eye Disease,” “IGF-1R inhibition,” “adverse effects,” and related keywords. Studies were selected based on their relevance to TED pathogenesis, mechanistic pathways, and clinical safety of teprotumumab. Articles were included if they (1) were published in peer-reviewed journals, (2) reported clinical or mechanistic findings on teprotumumab, and (3) were available in English. Studies were excluded if they were editorials, commentaries, or lacked primary data. Since this is a narrative review rather than a systematic review, we did not strictly follow PRISMA guidelines but focused on synthesizing key findings from available literature.

By shedding light on these aspects, this review provides a more holistic understanding of teprotumumab’s safety profile, guiding clinicians in making more informed treatment decisions and improving patient outcomes.

## 2 IGF-1R and TSHR activation drives TED pathogenesis and complicates treatment

The pathogenesis of TED involves a multifaceted interplay of genetic, immune, and environmental factors. It’s crucial to note that orbital fibroblasts (OFs), a type of connective tissue cell found around the eyes, are the primary contributors to inflammation and swelling in TED. The thyroid-stimulating hormone receptor (TSHR) and IGF-1R are common antigens in TED. The signaling pathways of TSHR and IGF-1R collaboratively initiate and promote the development and progression of TED.

### 2.1 Orbital fibroblasts as effector cells in TED

OFs are integral effector cells in TED, contributing significantly to this condition’s characteristic soft tissue swelling. OFs are typically located in the interstitial spaces between muscle fibers, orbital fibers, and connective tissue, playing a critical role in TED’s pathogenesis and progression (10). These cells are divided into CD34+ and CD34- subtypes, with CD34+ OFs absent in healthy individuals’ orbits but comprising approximately 30% of OFs in TED patients (11). CD34+ OFs exhibit significantly higher TSHR and thyroid peroxidase mRNA levels than CD34- OFs (12, 13). Studies have shown that activated OFs in TED patients respond robustly to pro-inflammatory cytokines and growth factors, secreting elevated levels of interleukins (IL-1α, IL-1β, IL-6, IL-8), macrophage chemotactic protein-1 (MCP-1), and transforming growth factor-β (TGF-β), thereby exacerbating inflammation (14). Additionally, OFs can proliferate and differentiate into myofibroblasts and adipocytes, produce excessive glycosaminoglycans, and promote adipogenesis. They also actively interact with mononuclear cells, producing chemokines and cytokines that perpetuate orbital inflammation. Consequently, OFs are crucial in initiating and maintaining TED-related inflammation and tissue remodeling (15).

### 2.2 IGF-1R and TSHR as autoantigens in the pathogenesis of TED

Recent research has focused on the involvement of IGF-1R and TSHR in the pathogenesis of TED. TSHR is TED’s most common pathogenic antigen, expressed in orbital connective tissues and extraocular muscles. While its expression is low in cultured OFs and normal orbital fat tissue, it is significantly elevated in the orbital tissues of TED patients (16). The expression level of TSHR correlates closely with the clinical activity and severity of TED (17). IGF-1R, a receptor tyrosine kinase, is broadly expressed in human cells, including OFs, and plays a crucial role in immune regulation, making it a therapeutic target in autoimmune diseases (18).

In female mouse models of TED, OFs exhibit high levels of IGF-1R expression and increased adipogenesis. Compared to control animals, IGF-1 stimulation significantly elevates hyaluronic acid secretion through IGF-1R activation (19). In TED patients, OFs express high levels of IGF-1R, which regulate lymphocyte trafficking, hyaluronic acid production, and fat accumulation and define the phenotypes and functions of T and B lymphocytes (20, 21). Furthermore, IgG from Graves’ disease patients activates IGF-1R, leading to inappropriate expression of inflammatory factors, hyaluronic acid production, and OF activation (22). These studies indicate that IGF-1R is an autoantigen in TED, contributing to the disease’s pathogenesis and immune system modulation. The IGF system, a complex network comprising ligands (IGF-1, IGF-2, and insulin), receptors (IGF-1R, IGF-2R, and insulin receptor), and IGF-binding proteins, is a fascinating study area. IGF-1 and IGF-2, the primary players, exert their effects through IGF-1R (23), a transmembrane glycoprotein dimer (heterotetramer) that shares structural similarities with the insulin receptor. The mature IGF-1R protein’s extracellular region, which includes elements of the α and β subunits, is involved in ligand binding. In contrast, the intracellular region contains the tyrosine kinase domain of the β subunit (24). IGF-1R, a receptor tyrosine kinase family member, is widely expressed in various human tissues and participates in numerous physiological processes such as growth and development, metabolism, energy consumption, and immune surveillance (25, 26).

At physiological ligand concentrations, IGF-1R undergoes significant structural rearrangement upon binding a single ligand and exerts its effects through two significant signaling pathways:

- The phosphatidylinositol 3-kinase (PI3K)-AKT/mammalian target of rapamycin (mTOR) pathway primarily controls metabolic functions.
- The Src homology 2 domain-containing transforming protein (SHC)-Ras-mitogen-activated protein kinase (MAPK) pathway mainly regulates mitosis-related functions such as cell growth and differentiation.

Studies have shown that the stimulating TSHR monoclonal antibody M22 in TED fibroblast tissues can directly activate TSHR and trigger IGF-1R signaling, immediately increasing hyaluronic acid secretion (27). Recent research confirms that stimulating TSHR antibodies can induce IGF-1R phosphorylation and activate both TSHR and IGF-1R signaling pathways. ANTAG3, a selective TSHR antagonist, can inhibit both IGF-1R-dependent and IGF-1R-independent pathways, proving more effective than the IGF-1R inhibitory antibody 1H7 in suppressing TED-Ig-induced hyaluronic acid secretion (28). These findings suggest that activating or blocking TSHR can simultaneously trigger or inhibit IGF-1R signaling pathways, ultimately affecting hyaluronic acid synthesis. TSHR and IGF-1R co-localize on the membranes of orbital cells, forming a protein complex that interacts physiologically and functionally (29). This suggests that the signaling pathways of IGF-1R and TSHR may overlap and interact, jointly contributing to the pathogenesis of TED.

## 3 Safety profile of teprotumumab for treating TED

### 3.1 Risk of hearing impairment with teprotumumab in treating thyroid eye disease

**Figure 1:**
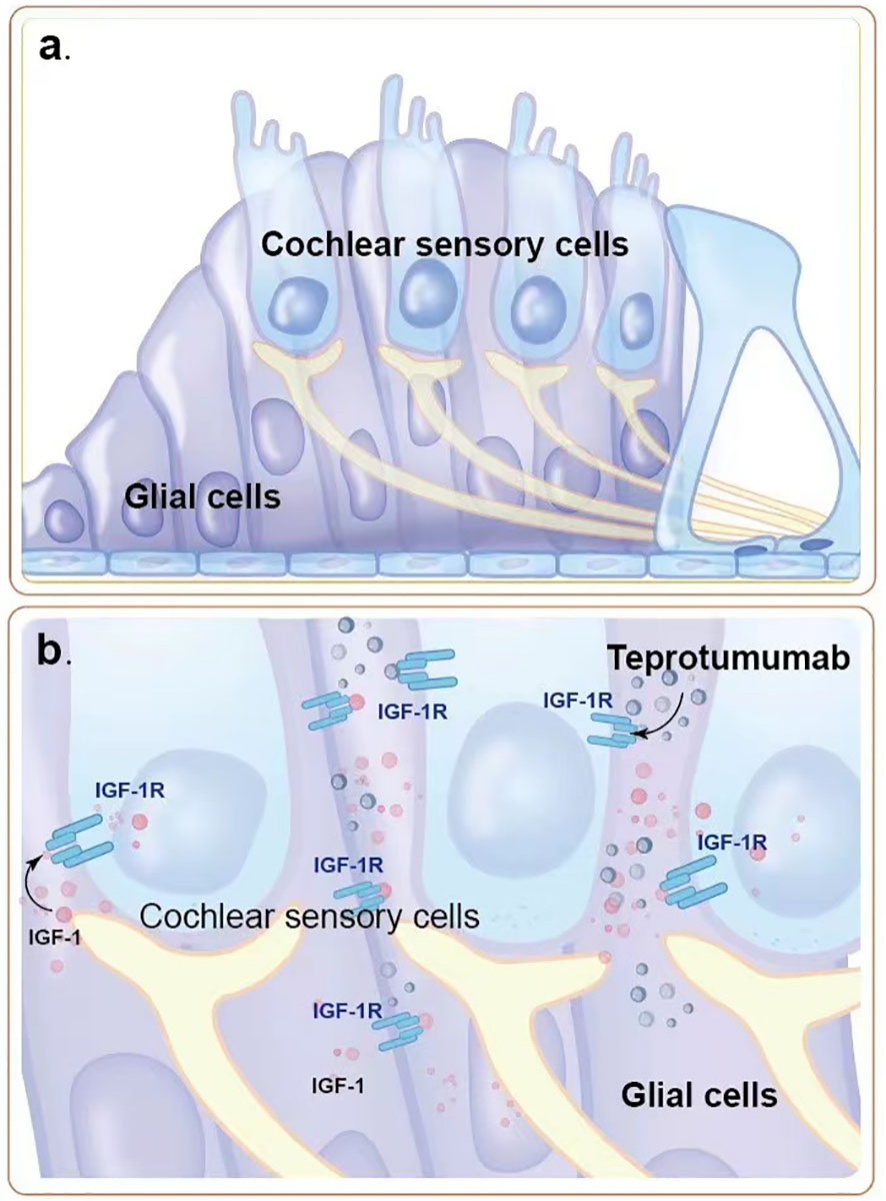
The role of IGF-1R signaling pathway in cochlear hair cells. **(a)** Normal structure and function of cochlear sensory cells and glial cells. IGF-1 binds to IGF-1R on cochlear sensory cells, activating intracellular signaling pathways that promote cell survival and proliferation, thereby maintaining the normal function of the auditory system. **(b)** Teprotumumab binds to IGF-1R, blocking IGF-1 from binding to its receptor, inhibiting intracellular signaling pathways. This leads to damage and reduction of cochlear sensory cells and glial cells, resulting in hearing impairment.

**Figure 2:**
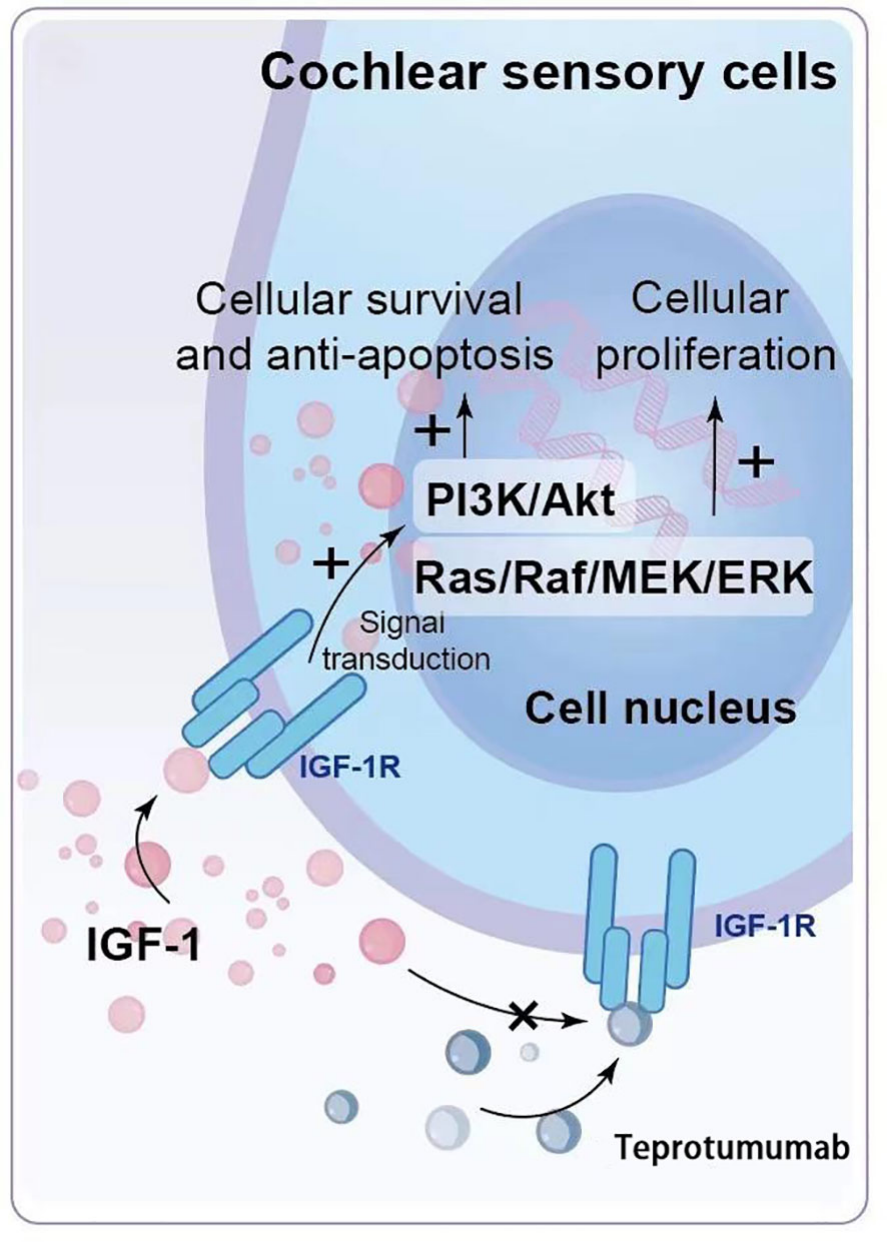
Mechanism of Hearing Impairment Induced by Teprotumumab. IGF-1 binds to IGF-1R on cochlear hair cells and supporting cells, activating intracellular signaling pathways such as PI3K/Akt and Ras/Raf/MEK/ERK, which are crucial for cell survival, proliferation, and functional maintenance. Teprotumumab binds to IGF-1R, blocking IGF-1 from activating these pathways, leading to impaired cell survival and proliferation, and ultimately resulting in hearing impairment.

Among the adverse reactions to teprotumumab, hearing impairment is particularly concerning. Teprotumumab, a biological agent that inhibits the IGF-1 pathway by targeting IGF-1R, disrupts critical signaling pathways in cochlear cells that rely on IGF-1 signaling for survival and proliferation (**Figures 1a, b**, **2**). The PI3K/Akt and Ras/Raf/MEK/ERK pathways, typically activated by IGF-1, support cochlear cell function. By blocking IGF-1’s interaction with IGF-1R, teprotumumab disrupts these pathways, impairing cochlear cell survival and contributing to hearing loss.

Hearing impairment associated with teprotumumab appears to involve both sensorineural and conductive components. Sensorineural impairments are linked to the inhibition of IGF-1 signaling in cochlear cells, while conductive impairments are likely due to dysfunction in the Eustachian tube, typically reversible (30, 31).

In clinical trials, approximately 10% of patients receiving teprotumumab reported hearing-related adverse events. In phase II and III trials for TED, 8 cases (9.5%) of hearing-related adverse reactions were reported in the teprotumumab group (0 cases in the placebo group); in the extension study of the phase III trial (32), 5 cases (10.9%) were reported. These events included hearing loss (5 cases), deafness (3 cases), tinnitus (3 cases), and Eustachian tube dysfunction (2 cases). In the OPTIC trial, 12 patients (9.9%) reported hearing-related adverse events, most of which were mild and resolved after treatment discontinuation (33).

Findings from observational studies highlight the importance of baseline hearing conditions. In a prospective study, 22 out of 27 patients (81.5%) receiving teprotumumab reported new subjective ear symptoms, but only 45.5% of those with hearing loss achieved full recovery (34). Importantly, Sears et al. (34) found that 46% of patients with post-treatment hearing issues had baseline hearing impairments.

Additionally, Douglas et al. (35) demonstrated that patients without baseline hearing dysfunction did not develop significant hearing impairment from teprotumumab treatment, underscoring the need for careful monitoring in high-risk populations.

Objective audiometric assessments, as highlighted by Keen et al. (36) and Douglas et al. (35), are critical for accurately evaluating the incidence and severity of hearing-related adverse effects. Sears et al. (34) noted that relying on subjective reports may introduce bias, making objective assessments more reliable.

Teprotumumab-related hearing impairment may also be associated with other disabling diseases, including cognitive decline, dementia, and depression (37). Research indicates that Graves’ Disease (GD) patients have a higher risk of hearing impairment, with studies showing IGF-1 deficiency in some GD patients (32, 38, 39). Several published cases have demonstrated persistent and potentially permanent sensorineural hearing loss related to teprotumumab (40, 41).

The U.S. prescribing label for TEPEZZA^®^ (teprotumumab) recommends conducting hearing evaluations before, during, and after treatment, emphasizing the importance of monitoring high-risk patients (42).

### 3.2 Hyperglycemia and blood glucose fluctuations induced by teprotumumab

**Figure 3:**
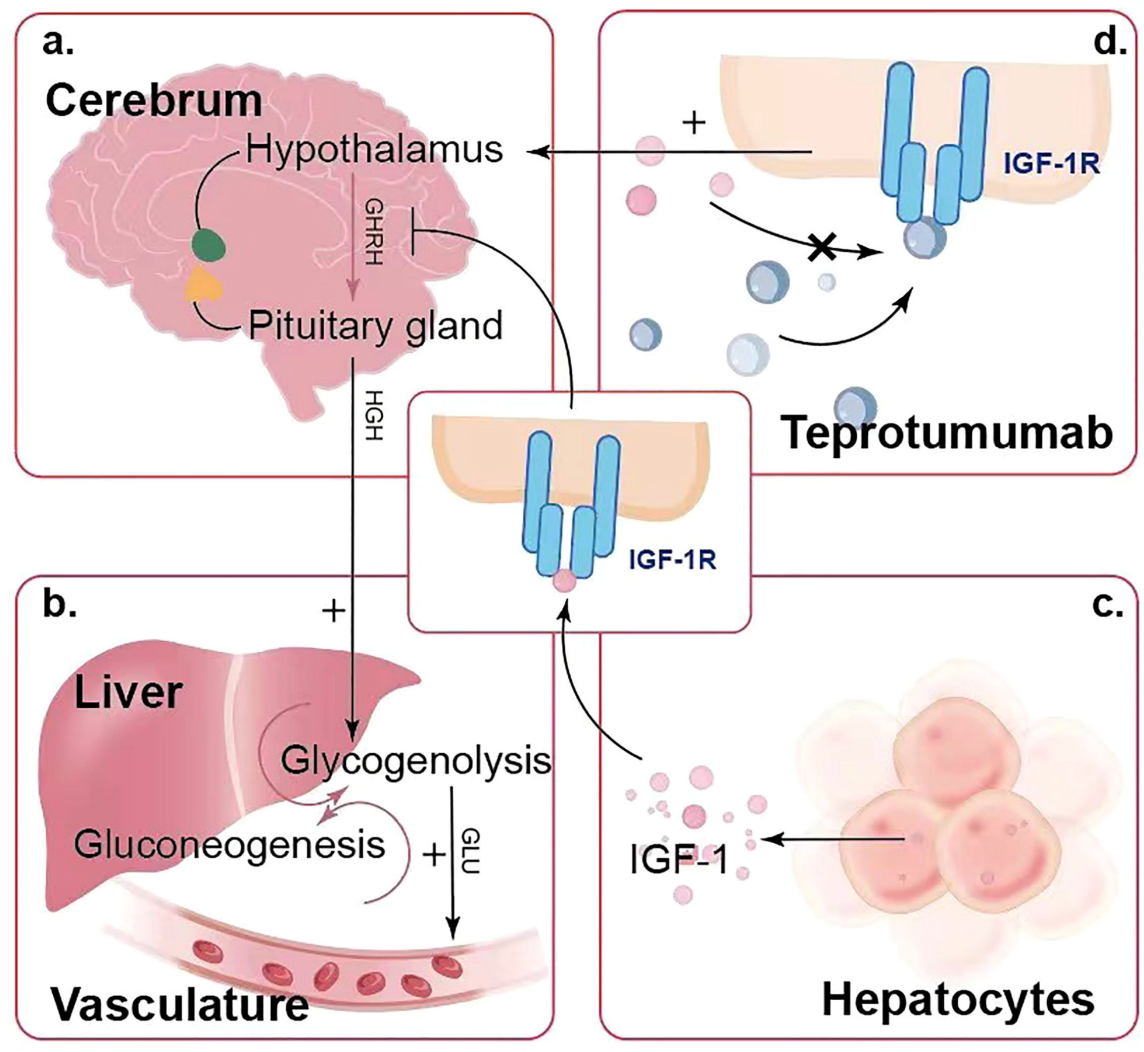
Mechanism of Teprotumumab-Induced Blood Glucose Fluctuations and Hyperglycemia. **(a)** The hypothalamus and pituitary gland regulate growth hormone secretion, which is feedback-regulated by IGF-1 levels. **(b)** Elevated growth hormone levels promote glucose production in the liver through glycogenolysis and gluconeogenesis, leading to increased blood glucose levels. **(c)** Hepatocytes produce IGF-1, which plays a role in this feedback mechanism. **(d)** Teprotumumab binds to IGF-1R, inhibiting IGF-1 from binding and disrupting the negative feedback, leading to elevated growth hormone levels and resulting in hyperglycemia.

Teprotumumab treatment has been associated with significant blood glucose fluctuations in some patients. The hypothalamus and pituitary gland regulate the secretion of various hormones, including growth hormone, which plays a vital role in glucose regulation. Under normal conditions, IGF-1 binds to IGF-1R on cells in the hypothalamus and pituitary gland (**Figures 3a, c**), providing negative feedback to inhibit growth hormone secretion, thereby maintaining stable glucose levels. However, teprotumumab inhibits IGF-1R, disrupting this negative feedback mechanism and increasing growth hormone secretion. Elevated growth hormone promotes glucose production in the liver through glycogenolysis and gluconeogenesis (**Figure 3b**) and increases insulin resistance, making it harder for insulin to lower blood glucose levels. Through these mechanisms, teprotumumab induces blood glucose fluctuations and hyperglycemia in some patients, especially early in treatment (**Figure 3d**). This complex mechanism may explain why some patients experience significant blood glucose fluctuations after receiving teprotumumab treatment.

Some patients experience significant blood glucose fluctuations after receiving teprotumumab treatment, especially early in treatment (43). Dysregulation of the hypothalamic-pituitary axis may lead to elevated serum glucose levels. Inhibition of IGF-1R can reduce the feedback inhibition on growth hormone secretion, increasing growth hormone levels, glucose production, and insulin resistance. Lee et al. (44) suggested that hyperglycemia is an adverse reaction due to the partial homology between IGF-1R and the insulin receptor. The indirect impact on insulin receptor signaling might also play a role, as IGF-1R inhibitors block the insulin receptor, preventing insulin from binding to its receptor, thereby inhibiting cellular glucose uptake and leading to hyperglycemia (45).

In a recent study analyzing three clinical trials (46), among 121 TED patients receiving teprotumumab, 8 cases (6.6%) of hyperglycemia were reported in the treatment group compared to no cases in the placebo group. This incidence of hyperglycemia was notably higher in patients with pre-existing diabetes or impaired glucose tolerance. Additionally, among 10 patients with a history of diabetes or impaired glucose tolerance, 5 (50%) developed hyperglycemia-related adverse events. In contrast, among the 74 patients without glucose tolerance impairment, only 3 cases (4.1%) of hyperglycemia were observed. Most hyperglycemic events occurred in patients with diabetes or impaired glucose tolerance, and all cases were managed with adjustments to their diabetes medication or with additional glucose-lowering therapy. These findings highlight that teprotumumab’s hyperglycemia-related adverse effects predominantly affect those with pre-existing glucose metabolism issues, emphasizing the importance of regular blood glucose monitoring for high-risk patients, such as those with diabetes or impaired glucose tolerance (3). Shah et al. (47) reported a case of a 56-year-old female TED patient who developed hyperglycemia symptoms three weeks after the first infusion of teprotumumab, eventually leading to a hyperosmolar hyperglycemic state (HHS). This case underscores the potential for teprotumumab to induce significant hyperglycemia in high-risk patients and highlights the need for vigilant monitoring and prompt management in such cases. Furthermore, the drug manufacturer has clearly stated in the prescription information that the incidence of hyperglycemia is approximately 10% in TED patients receiving teprotumumab.

Monitoring blood glucose levels in patients receiving teprotumumab treatment is crucial, with particular attention to high-risk groups (patients with prediabetes, diabetes, the elderly, and high-risk populations). If necessary, antidiabetic medications should be used to control blood glucose levels. Before starting treatment, patients should undergo fasting blood glucose and HbA1c testing to assess their baseline glucose control. As Douglas et al. (48) recommended, close collaboration between ophthalmologists and endocrinologists is essential to address the potential risk of hyperglycemia, and patients with poorly controlled diabetes should aim to stabilize HbA1c levels before initiating teprotumumab treatment.

During treatment, it is essential to increase the frequency of blood glucose monitoring, adjusting based on baseline diabetes status and other risk factors. Patients are most susceptible to hyperglycemia early in the treatment course, as highlighted by the incidence data from controlled trials (49). Proactive measures should be taken to control hyperglycemia effectively, such as daily fasting and postprandial blood glucose monitoring during initial treatment weeks, with weekly follow-ups to adjust antidiabetic medication dosages as necessary (50). Future studies are warranted to further analyze the incidence and management of severe hyperglycemia and HHS in patients treated with teprotumumab.

### 3.3 Potential reactivation and exacerbation of inflammatory bowel disease

Research indicates an inverse correlation between serum IGF-1 levels and the severity of several autoimmune diseases, including rheumatoid arthritis and inflammatory bowel disease (IBD) (51–53). Although the etiology of IBD remains unclear, active IBD patients typically exhibit reduced serum IGF-1 levels (54), which may be secondary to gastrointestinal dysfunction and acquired growth hormone resistance (55). Under normal physiological conditions, IGF-1, stimulated by growth hormone, promotes cell proliferation, somatic growth, and cellular synthesis processes. IGF-1R, expressed on intestinal epithelial cells and smooth muscle cells, facilitates the regulation of growth and function of adjacent intestinal epithelial cells by IGF-1 derived from intestinal mesenchymal cells (56). In the gastrointestinal tract, IGF-1 is crucial for promoting mucosal proliferation and repair, preventing apoptosis, supporting mucosal barrier function, and mitigating inflammation (57–59). Thus, it is hypothesized that inhibiting IGF-1R could reduce IGF-1 levels in both serum and gastrointestinal tissues. The IGF-1 reduction caused by teprotumumab’s inhibition of IGF-1R may mimic conditions observed in IBD patients, potentially leading to similar gastrointestinal symptoms and signs. However, it should be noted that the current clinical trial data have not established a direct association between teprotumumab and IBD reactivation. The majority of reported diarrhea cases did not specify IBD as an underlying condition, suggesting that gastrointestinal adverse events associated with teprotumumab may not necessarily indicate IBD causation. As stated in the prescribing label (60), “Exacerbation of Preexisting Inflammatory Bowel Disease (IBD): Monitor patients with preexisting IBD for flare of disease; discontinue TEPEZZA if IBD worsens.” This underscores the potential risks of teprotumumab in IBD patients and the importance of monitoring but does not confirm causation between teprotumumab and IBD exacerbation.

Clinical data on diarrhea incidence highlight a 13% occurrence rate among patients receiving teprotumumab. In the phase II trial for TED, 14% (6/43) of patients treated with teprotumumab experienced diarrhea, including two cases where patients with a history of colitis had significant disease exacerbation during treatment (3). Consequently, IBD patients were excluded from the phase III trial, and the FDA was advised to list IBD as a precautionary contraindication rather than a strict contraindication. Additionally, spontaneous case reports, such as that by Ashraf et al., described a TED patient with a family history of IBD who developed new-onset IBD during teprotumumab treatment (61). Similarly, Safo et al. (62) reported a case involving a 46-year-old female who developed bloody diarrhea and urgency following five infusions of teprotumumab, which ultimately led to a diagnosis of ulcerative colitis (UC) confirmed by colonoscopy and biopsy. However, it is essential to note that individual case reports cannot establish a causal link between teprotumumab and IBD development, as spontaneous reports alone lack the statistical strength required for causation.

Based on these observations, it is critical to exercise caution with teprotumumab in patients with a history of IBD or a family history of autoimmune diseases. Thorough screening of patients’ autoimmune disease history, particularly family history, is not just recommended but essential prior to initiating treatment. Patients identified with a personal or family history of IBD should be informed of the potential risks associated with the medication. During treatment, close monitoring for acute gastrointestinal symptoms, such as gastrointestinal bleeding, abdominal pain, and diarrhea, is essential. If signs of IBD exacerbation, such as persistent severe diarrhea or hematochezia, occur, gastroenterological evaluation is strongly advised, and consideration should be given to discontinuing teprotumumab infusion.

### 3.4 Muscle cramps associated with IGF-1R inhibition

Research indicates that IGF-1 is crucial for skeletal muscle growth, repair, and prevention of degradation (63, 64). Inhibiting IGF-1R can lead to muscle cramps. Precisely, IGF-1 activates the PI3K/Akt pathway. Upon binding to its receptor IGF-1R, IGF-1 stimulates the intracellular PI3K/Akt signaling cascade. This pathway promotes protein synthesis and enhances cellular growth and proliferation by upregulating the activity of the mammalian target of rapamycin (mTOR). Additionally, it inhibits the FoxO (Forkhead Box O) transcription factors responsible for protein degradation. Furthermore, IGF-1 activates muscle satellite cells, which can differentiate into myoblasts following muscle injury, thus promoting muscle repair and regeneration (65).

In phase II and III trials for TED, muscle cramps were the most common adverse reaction to teprotumumab (25%) (3). Most cases were mild, with five moderate cases reported, but none required treatment discontinuation. The lower limbs were the most frequently affected area, with no clinically relevant laboratory abnormalities detected. It’s reassuring to know that treatment measures for muscle cramps may include massage, Epsom salt baths, or supplementation with magnesium, calcium, and potassium (66). Adequate hydration and using muscle relaxants are also effective in alleviating symptoms when necessary.

### 3.5 Management and characteristics of infusion reactions to teprotumumab

Infusion reactions induced by teprotumumab, although not fully understood, are believed to arise from cytokine release following antibody-antigen interactions (67). As with many human monoclonal antibodies, infusion reactions with teprotumumab are typically mild, though severe cases have been observed. In the Phase III trial (4), two patients experienced infusion reactions; one reaction occurred during the initial infusion, which was managed effectively within two hours, and the infusion was discontinued. After premedication and a reduced infusion rate, another patient completed the trial without further severe reactions. Both cases underscore the need for a well-prepared clinical environment to address potential infusion-related adverse events.

Management protocols recommend immediate cessation of infusion upon recognition of a reaction, followed by assessment of vital signs and monitoring of airway status. According to recent recommendations by Kang et al. (68), once symptoms subside, premedication with antihistamines, acetaminophen, and corticosteroids, along with a slower infusion rate, may reduce the severity of subsequent reactions. In moderate reactions, gradual resumption of the infusion may be considered, although all patients should be closely monitored.

For severe reactions, rapid intervention is paramount. Patients should receive oxygen supplementation and, if necessary, bronchodilators, epinephrine, antihistamines, and corticosteroids. Emergency response protocols recommend that infusion centers maintain ready access to resuscitation equipment and that staff are trained to recognize and manage severe infusion reactions promptly. This approach minimizes risk and enhances patient safety during treatment.

Given the potential for infusion reactions, particularly during the initial infusion or within 90 minutes of administration, it is essential to inform patients of these risks beforehand. Patients should be educated to recognize early symptoms, such as flushing, nausea, headache, or shortness of breath, and to notify healthcare staff immediately if symptoms occur. Clear communication with patients allows for swift intervention, minimizing the risk of severe outcomes.

## 4 Other adverse reactions reported in literature

Studies indicate that teprotumumab is associated with several additional adverse effects, including cognitive decline, thyroid dysfunction, alopecia, nausea, and fatigue. In a Phase II trial (4), a 61-year-old male patient experienced multiple transient cognitive changes. Hoang et al. (69) also reported a 76-year-old male TED patient who developed rapidly progressive cognitive decline after four infusions of teprotumumab. Although the exact mechanism remains unclear, the critical role of IGF-1 and its pathways in central nervous system development and function provides a rationale for the link between IGF-1R inhibition and cognitive decline (70). Additionally, Yu et al. (71) reported a 41-year-old female TED patient who developed significant thyroid dysfunction after two months of teprotumumab treatment. Research has shown that IGF-1R forms a complex with TSHR, mediating the effects of thyroid-stimulating hormone and GD immunoglobulins in orbital fibroblasts and thyroid epithelial cells (8). In mice, IGF-1R gene knockout in the thyroid reduced circulating thyroid hormones and increased thyroid-stimulating hormone levels (72). Thus, this medication may inhibit thyroid function in TED patients, and close monitoring of thyroid hormone levels during treatment is recommended. Furthermore, the most common adverse reactions in Phase II and III trials included alopecia (13%), nausea (17%), and fatigue (10%) (49).

Overall, continued pharmacovigilance and potential additional research are needed for adverse reactions related to teprotumumab. As our understanding of the drug evolves, further in-depth studies are required to explore its dose range, variable concentrations, infusion frequency, and duration of treatment. Teprotumumab, being a new drug with limited clinical research and safety data, requires a broader sample size and more comprehensive clinical data before widespread clinical application. Although rare, the adverse reactions should be carefully monitored. The mechanisms behind teprotumumab’s systemic side effects remain unclear and are poorly understood. In clinical practice, ophthalmologists must prioritize drug safety, remain vigilant for complications, and address them promptly to minimize patient harm. Further, extensive research on the drug’s efficacy, safety, optimal dosing, and usage is crucial to determine whether teprotumumab can become a new first-line treatment for TED.

## 5 Discussion

The current therapeutic strategies for TED are tailored to the disease’s severity, activity, and clinical manifestations. Treatment options for active, moderate-to-severe TED include corticosteroids, retrobulbar radiotherapy, orbital decompression surgery, and other immunosuppressive or biological therapies. As non-specific immunomodulators, Corticosteroids are recommended as the first-line treatment for active TED. While corticosteroids can effectively reduce inflammation and local congestion in some patients, their efficacy in ameliorating proptosis and diplopia is limited (27). Even with the highest cumulative dose (7.47 g), corticosteroids fail to improve TED outcomes (28) significantly.

Rituximab, a chimeric monoclonal antibody targeting the CD20 surface antigen on B cells, has demonstrated superiority over methylprednisolone in reducing the Clinical Activity Score (CAS), yet it remains ineffective for proptosis (73). Tocilizumab, a humanized monoclonal antibody against the IL-6 receptor, has significantly improved CAS and soft tissue signs in active, moderate-to-severe TED. However, it does not ameliorate proptosis and is associated with higher infection rates and headaches (74, 75). Thus, the current treatment for active TED is predominantly limited to anti-inflammatory and immunosuppressive drugs, which exhibit limited efficacy and do not significantly improve clinical outcomes. Once TED reaches a stable phase, elective surgical interventions become necessary; however, these procedures carry inherent risks, including unpredictable outcomes and the potential for disease reactivation.

Recent studies have increasingly confirmed the pivotal role of IGF-1R as an autoantigen in the pathogenesis of TED, mediated through immune-inflammatory mechanisms and interactions with TSHR. Targeting IGF-1R offers a potential mechanistic approach to TED therapy. Teprotumumab, the first therapeutic-specific IGF-1R antagonist, provides etiological and targeted treatment advantages markedly superior to traditional TED therapies. Clinical research has demonstrated that teprotumumab significantly reduces the activity and severity of TED, exhibiting a favorable safety profile, minimal side effects, sustained therapeutic effects, and improved quality of life for patients. The efficacy of teprotumumab is reinforced by its favorable benefit-risk ratio, suggesting its potential as a first-line treatment for TED.

However, despite its promising benefits, several safety concerns remain. Teprotumumab has been associated with adverse effects such as hearing impairment, hyperglycemia, and potential exacerbation of pre-existing inflammatory bowel disease. Given these risks, clinicians should take a proactive approach in patient selection and monitoring to mitigate these complications.

To optimize clinical outcomes and ensure patient safety, we propose the following clinical recommendations for the use of teprotumumab in TED treatment:

**a. Pre-treatment Screening**: Patients should undergo baseline audiometric testing, fasting blood glucose assessment, and a thorough medical history review for pre-existing diabetes or inflammatory bowel disease. This will help identify high-risk individuals who may require closer monitoring.
**b. Hearing Loss Monitoring**: Given that teprotumumab-related hearing impairment has been reported in up to 10% of treated patients, audiometric testing should be performed before treatment initiation, at mid-treatment, and post-treatment. Patients with pre-existing hearing dysfunction should be counseled on the potential risk of exacerbation.
**c. Blood Glucose Management**: As teprotumumab can induce hyperglycemia, patients with diabetes or impaired glucose tolerance should have their blood glucose levels carefully monitored. In high-risk patients, collaboration with an endocrinologist may be beneficial. Adjustments in diabetes medications should be considered if significant hyperglycemia occurs during treatment.
**d. IBD Risk Assessment:** Although data on teprotumumab-induced IBD exacerbation are limited, patients with a history of Crohn’s disease or ulcerative colitis should be closely monitored for gastrointestinal symptoms. If severe exacerbations occur, discontinuation of teprotumumab should be considered.

Future research should focus on identifying predictive biomarkers for adverse effects, refining risk stratification models, and optimizing dosing regimens to maximize therapeutic benefits while minimizing toxicity. Additionally, long-term follow-up studies are needed to evaluate the durability of teprotumumab’s therapeutic effects and its impact on TED progression over time.

Currently, teprotumumab is the only IGF-1R antagonist approved by the FDA for TED treatment, indicating promising prospects for broader clinical acceptance by hospitals and patients. However, further real-world studies and pharmacovigilance efforts are essential to better characterize its long-term safety and efficacy profile. Clinicians should remain vigilant for potential complications and adopt a multidisciplinary approach to TED management, ensuring comprehensive patient care.

In conclusion, while teprotumumab represents a significant advancement in TED management, it is imperative to balance its efficacy with safety considerations. By incorporating standardized screening, monitoring, and multidisciplinary collaboration, clinicians can enhance treatment success while minimizing the risks associated with teprotumumab therapy.
